# Bibliometric Analysis of Veterinary Communication Education Research over the Last Two Decades: Rare Yet Essential

**DOI:** 10.3390/vetsci9060256

**Published:** 2022-05-27

**Authors:** Zih-Fang Chen, Yi-Hsin Elsa Hsu, Jih-Jong Lee, Chung-Hsi Chou

**Affiliations:** 1 Zoonoses Research Center, School of Veterinary Medicine, National Taiwan University, Taipei 10617, Taiwan; zifung@gmail.com; 2 Executive Master Program of Business Administration in Biotechnology, Taipei Medical University, Taipei 11031, Taiwan; 3 School of Healthcare Administration, Taipei Medical University, Taipei 11031, Taiwan; 4 Institute of Veterinary Clinical Science, School of Veterinary Medicine, National Taiwan University, Taipei 10617, Taiwan; jacklee@ntu.edu.tw

**Keywords:** veterinary education, communication, veterinarians, clients, bibliometrics, VOSviewer, Web of Science Core Collection database

## Abstract

Research of veterinary communication education is a relatively rare but important field, and its importance has been increasingly noticed recently. This study aims to describe the existing veterinary education research literature by adopting the systematic bibliometric approach. We conducted a comprehensive literature exploration on worldwide veterinary education and veterinary communication education publications in the Web of Science Core Collection database from 1 January 2000 to 31 December 2021. VOSviewer and EXCEL were used to identify trends and patterns in characteristics of the publications, including author affiliations and countries, and the publishing journals. Based on our search criteria, in the past 22 years, there have been 6006 veterinary education publications with 101 publications in 2000, 684 publications in 2021 (577% increase), and 677 communication-related publications with 9 publications in 2000, 107 publications in 2021 (1189% increase). The VOSviewer results indicate that both the United States and England were the most vigorous countries with close collaboration. Our results show the publication quantity has been increasing at a sharp slope rate over the past twenty years, which indicates the importance and growth of veterinary education and the veterinary communication education research field, and identifies the international collaborations among countries and institutions.

## 1. Introduction

Research of veterinary communication education is a relatively rare but important field. Veterinary communication is one of the most common barriers to veterinary care, yet there is only limited literature published in this field [1,2]. The earliest research on veterinary communication education was published in the early 1910s by Veranus Alva Moore, a bacteriologist, veterinary pathologist, professor, and Dean of the New York State Veterinary College at Cornell University, with the title of “American veterinary education and its problems” [3]. Professor Moore indicated that training clients, who own animals, was an important stone placed in the foundation for more efficient veterinary education, and effective veterinarian–client communication was key to training results.

Client–veterinarian communication has recently been found to be one of the most focusing themes [4]. The client–veterinary interaction is delineated in an initial greeting, history taking, performing physical examination, explaining diagnosis, offering treatment options, and closing the interaction. A study by the National Unit for the Advancement of Veterinary Communication Skills (NUVACS) showed that more than 80% of complaints brought against veterinarians revolve around problems of poor communication [5]. The problems associated with communication between veterinarians and animal owners and unforeseen random situations are the general causes of conflict, and conflict in the course of work is most often experienced by young veterinarians [6].

Research on and the teaching of communication as an essential clinical skill is a more recent development in veterinary medicine [5]. The importance of veterinary communication education has been increasingly noticed in these two decades [7]. For example, based on our search criteria with the keyword “veterinary education”, there were 6006 veterinary education publications (VEPs) in the Web of Science Core Collection (WoSCC) database in the last 22 years, yet only 677 (11.27%) publications with the keyword “veterinary communication education” (veterinary communication education publications, VCEPs). Although there are rare publications in the communication field, it contains many essential and important research topics, such as veterinarians communicating with farmers about management and control disease [8,9,10,11], the decision making related to herd health [12], communicating with pet owners about canine behavior training [13], the perceptions of the monetary aspects of veterinary care [14], veterinarian–client communication [15,16], and work-related stress in the veterinary profession [6,17].

Bibliometric analysis is a scientific methodology that can be used for descriptive purposes, which combines descriptive statistical methods with information visualization technology to identify core research characteristics of a specific area and its evolving trends in the worldwide research literature, or for providing a basis for evaluation, such as evaluating research outputs, influences, or other factors [18,19,20]. Pritchard (1969) identified that “bibliometrics” is an alternative to “statistical bibliography”, and bibliometric analyses can provide an empirical view of a field through the investigation of information about the research publications [21]. In the past two decades, there have been few bibliometric publications in the veterinary field. The topics of those bibliometric publications include analysis of a specific veterinary journal [22,23,24,25], or analysis of specific research fields, such as parasitological [26], antimicrobial resistance in wildlife [27], interdisciplinary and collaborative publications [28], gut health [29], and canine leishmaniasis [30].

However, there were no related systematic literature pattern analysis about communication education in veterinary medicine in the past two decades. This study aims to (1) describe the existing veterinary education research literature by retrospectively identifying trends and patterns in characteristics of the publications, including author affiliations and countries and the publishing journals, by adopting the bibliometric review approach; and (2) investigate the characteristics of both veterinary education publications (VEPs) and the rare yet essential subsets in VEPs, veterinary communication education publications (VCEPs).

## 2. Materials and Methods

We conducted a comprehensive literature exploration in the WoSCC database at National Taiwan University, Taiwan. This database was chosen due to the journals within the database having been selected for inclusion based on high impacts in the respective fields. To understand the world research trends over the past 22 years, we collected all the related research published from 1 January 2000 to 31 December 2021. In the WoSCC database, “Topic” was one of the most common fields used for database searching and it searches for the entered term in Titles, Abstracts, Author keywords, and Keyword Plus fields of records in the WoSCC database. We used the following search strategy for identifying the related publication in the WoSCC. We searched for veterinary or veterinarian with any of the following terms: education, training, or learn by topic, and the time period was limited from 2000 to 2021. The searching strategy used was: (veterinary or veterinarian) (Topic) and (education, training, or learn) (Topic) in the Year 2000–2021. Additionally, the subset on communication was searched by adding the communication by topic in the same period of time.

Data searched from the WoSCC were downloaded for more detailed analysis, including the author names and their address, the years, titles, abstracts, citations, and research area of the publications, and the published journals related information, including impact factors and rankings. Summary statistics about the publications, such as the publication year, countries, and research areas were gathered through the WoSCC interface. The Microsoft Excel analysis tool was used to aggregate data downloaded from WoSCC and draw related figures, including the geographic-distribution world map.

Software was available for bibliometric analysis, such as VOSviewer (Leiden University’s Centre for Science and Technology Studies, Leiden, The Netherlands), which has recently been widely employed to conduct bibliometric research. The VOSviewer was used to visualize the analysis results on identifying the clusters of network maps on authorship and institution collaboration. VOSviewer is a publicly available tool. Researchers from Leiden University in the Netherlands, van Eck and Waltman, presented VOSviewer, a freely available computer program developed for constructing and viewing bibliometric maps in 2010 [31]. The VOSviewer Manual written by Nees Jan van Eck and Ludo Waltman at the University Leiden in 2022 [32] describes the software and applications in detail. VOSviewer is a software tool used for creating maps based on network data and for visualizing and exploring these maps. The functionality of VOSviewer can be summarized as follows: creating maps based on network data, visualizing and exploring maps. VOSviewer has been primarily intended for analyzing bibliometric networks, however, it can in fact be used to create, visualize, and explore maps based on any type of network data [32]. VOSviewer has been developed in the Java programming language and can run on most operating system platforms. VOSviewer can be downloaded from www.vosviewer.com (accessed on 1 January 2022) for free.

## 3. Results

### 3.1. Quantity of Publications

**Figure 1:**
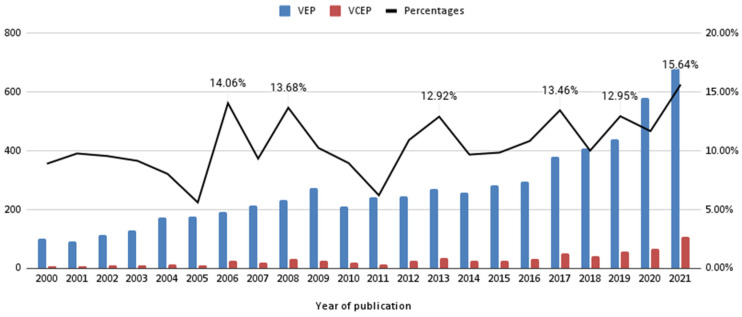
Yearly numbers of veterinary education publications (VEPs), veterinary communication education publications (VCEPs), and the percentages of VCEPs/VEPs (2000 to 2021).

Based on our search criteria, there have been 6006 veterinary education publications (VEPs) from 2000 to 2021. As shown in Figure 1, there were 101 VEPs (blue-color column in Figure 1) in 2000 and 684 publications in 2021, with a 577% increase.

Among these collected publications based on our search criteria, communication-related publications (denoted as “veterinary communication education publication”, VCEPs) were 677, which was 11.27% of the total publications from 2000 to 2021. As shown in Figure 1, there were 9 VCEPs (red-color column in Figure 1) in 2000 and 107 publications in 2021, with a 1189% increase. The black line shown in Figure 1 represents the percentages of VCEPs over VEPs from 2000 to 2021.

### 3.2. Highly Cited Publications

Highly cited articles, known as “citation classics”, are often acknowledged as highly influential in the field [33]. The 10 most highly cited publications in veterinary education publications from 2000 to 2020, including 7 original articles and 3 reviews, are shown in Table 1. The top highly cited publication was by Sike (2016) with 1088 citations [34]. Among these publications, communication-related highly cited top 10 publications, including 8 original articles and 2 reviews, are shown in Table 2. We showed the 10 top-cited publications and the details of these publications, including author, title, and published year, are summarized in Table 1 and Table 2.

### 3.3. Publications Titles

Our search terms identified a total of 872 SCI/SSCI journals (also named “publication titles” in WOSCC), which have published research manuscripts in the veterinary education field (*n* = 6006). The top 10 journals most published and their related characteristics, including impact factors and rankings in 2020, are listed in Table 3. The Journal of Veterinary Medical Education has topped the list with 1191 publications (19.83%) with an impact factor of 1.027. The Journal of The American Veterinary Medical Association (JAVMA) has been ranked second with 263 publications (4.38%), and Veterinary Record ranked third with 257 publications (4.28%). These journals, especially the Journal of Veterinary Medical Education, were undoubtedly of importance to this field. It is noteworthy that the publication numbers of other journals in this field were relatively small. Among 872 journals, there were only 7 journals (0.8%) that published more than 100 research publications, and 519 journals (59.52%) published only 1 publication.

In the subset of veterinary education publications (*n* = 677), 872 SCI/SSCI journals have published research manuscripts. The top 10 most published journals and their related characteristics, including impact factors and rankings in 2020, are listed in Table 4.

The Journal of Veterinary Medical Education topped the list with 215 publications (31.76%) with an impact factor of 1.027. Veterinary Record ranked second with 32 publications (4.73%), and Frontiers in Veterinary Science was ranked third with 30 publications (4.43%). These journals, especially the Journal of Veterinary Medical Education, were also undoubtedly of importance to this field. It is noteworthy that the publication numbers of other journals in this field were relatively small. Among 163 journals, there were only 6 journals (0.89%) that published more than the other 157 journals (347 and 330, respectively), and 90 journals (13.29%) published only 1 publication.

### 3.4. Countries’ Publications and the Collaborations

The 6006 veterinary education publications (VEPs) based on our search criteria collected from WOSCC from 2000 to 2021 were published by 152 countries, and 677 veterinary communication education publications (VCEPs) were published by 78 countries. The top 10 countries participating in VEPs and VCEPs are presented in Table 5. The top 10 countries participated in at least 170 studies related to VEPs and at least 14 related to VCEPs. Among them, the United States and England were the leading countries in both VEPs and VCEPs. The United States participated in the most studies in both areas, with 2159 (36.55%) and 296 (43.72%), respectively, followed by England with 777 (12.94%) and 115 (16.99%), respectively. The top third country was Australia in VEPs (460, 7.66%) and Canada in VCEPs (115, 16.99%).

**Figure 2:**
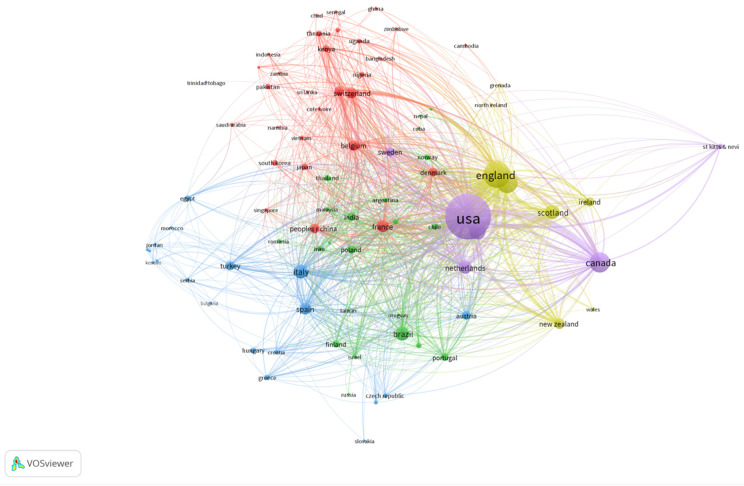
Country collaboration analysis of 6006 veterinary education publications (VEPs) from 2000 to 2021.

We adopted VOSviewer to analyze the co-authorship collaboration among the countries (Figure 2). The thickness of the lines indicates the scale of collaboration between the countries. The results show that both the United States and England are the most vigorous countries in this field, and these two countries also have a close collaboration.

A total of 152 countries contributed to veterinary education publications (VEPs) in the last 22 years. The top 5 countries/regions that published the most were as follows: USA (2195, 36.55%), England (777, 12.94%), Australia (460, 7.66%), Canada (435, 7.24%), and Germany (294, 4.90%).

**Figure 3:**
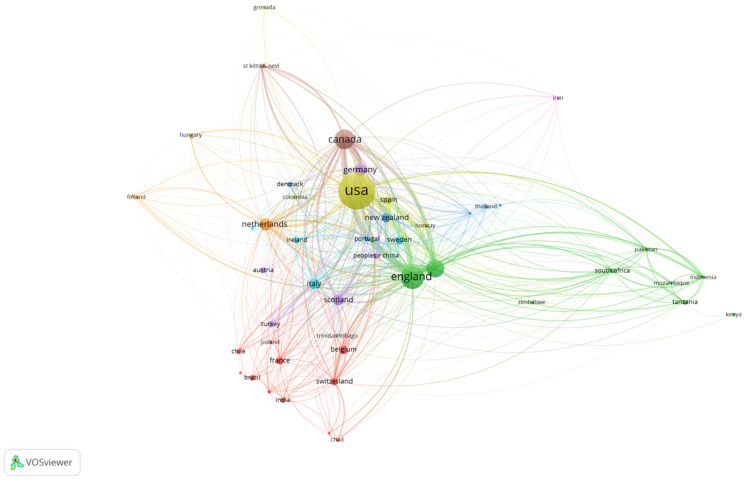
Country collaboration analysis of 677 veterinary communication education publications (VCEPs) from 2000 to 2021.

Regarding veterinary communication education publications (VCEPs), a total of 78 countries contributed to VCEPs in the last 22 years. The top 5 countries/regions that published the most were as follows: USA (296, 43.72%), England (115, 16.99%), Canada (84, 12.41%), Australia (71, 10.49%), and Germany (31, 4.58%). The VOSviewer visualized country co-authorship collaboration analysis is presented in Figure 3. The results also indicate that the United States is the most vigorously publishing country regarding the VCEPs, with close collaborations with lots of countries, including England and Canada.

### 3.5. Institutions

**Figure 4:**
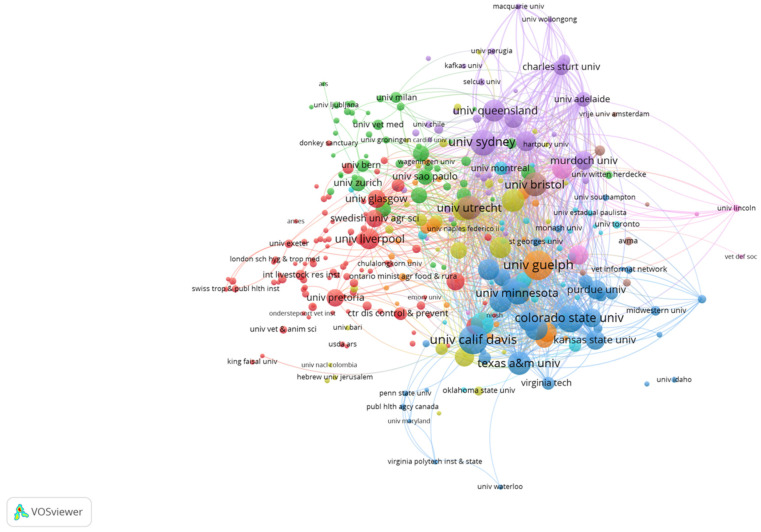
Institutional collaboration analysis of 6006 veterinary education publications (VEPs) from 2000 to 2021.

Based on our search criteria, the 6006 veterinary education publications (VEPs) were published by 4557 institutions and organizations to which all the authors belonged. The University of London had the greatest numbers of publications (228, 3.80%), followed by the University of London Royal Veterinary College (201, 3.35%), University of California System (192, 3.20%), University of Guelph (185, 3.08%), and University of California Davis (174, 2.90%). Institution collaboration analysis by VOSviewer of 6006 veterinary education publications (VEPs) from 2000 to 2021 is shown in Figure 4.

**Figure 5:**
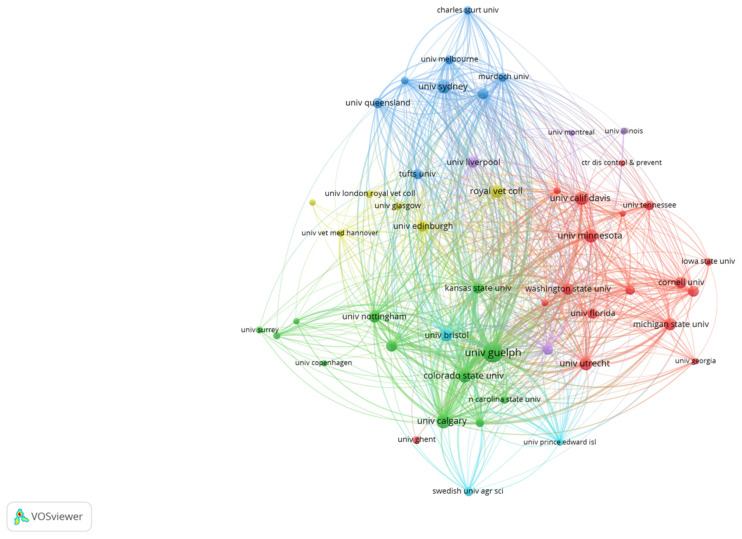
Institutional collaboration analysis of 677 veterinary communication education publications (VCEPs) from 2000 to 2021.

The 677 veterinary communication education publications (VCEPs) were published by 778 institutions and organizations to which all the authors belonged. The University of Guelph had the greatest numbers of publications (51, 7.51%), followed by the University of London (36, 5.30%), University of London Royal Veterinary College (36, 5.30%), University of Calgary (27, 3.98%), and University of California System (25, 3.68%). Institution collaboration analysis by VOSviewer of 677 veterinary communication education publications (VCEPs) from 2000 to 2021 is shown in Figure 5.

The results indicate that there are few major research networks of institutional collaboration in veterinary education publications (VEPs), including leading institutions in the United States, Australia, and the United Kingdom. However, the collaboration network of institutions of veterinary communication education publications (VCEPs) is comparatively small and sparse.

### 3.6. Authors

The 6006 veterinary communication publications collected based on our search criteria were published by 17,152 authors (not including Anonymous). The top three authors were as follows: Sarah Baillie from the University of Bristol in the UK with 44 publications (0.733%), Stephen May from the University of London Royal Veterinary College in the UK (38, 0.633%), and Cindy Adams from the University of Calgary in Canada (34, 0.566%). The 10th ranked author published 21 works.

The 677 veterinary communication education publications collected based on our search criteria were published by 2231 authors. The top three authors were as follows: Cindy Adams from the University of Calgary in the Canada with 32 publications (4.71%), Jason B. Coe from the University of Guelph in the Canada (20, 2.94%), and Jane R. Shaw from the Colorado State University in the United States (13, 1.91%). The interesting point is that these three top leading authors are all alumni of the University of Guelph. The 10th ranked author published 7 works. This result reveals that this is still a field with few publications and indicates that there is great potential for researchers to devote their studies to this field and become one of the top leading authors in the future.

**Figure 6:**
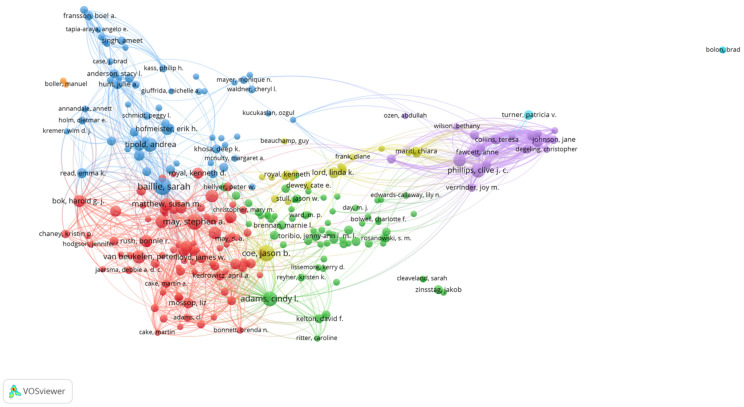
Author collaboration analysis of 6006 veterinary education publications (VEPs) from 2000 to 2021.

**Figure 7:**
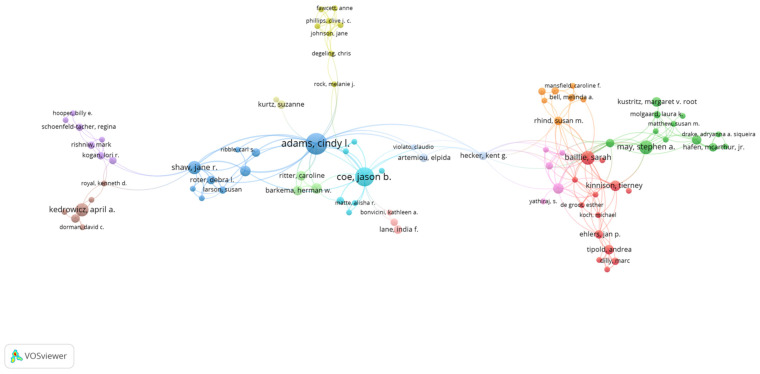
Author collaboration analysis of 677 veterinary communication education publications (VCEPs) from 2000 to 2021.

To further investigate the influential research teams and co-authorship networks, we performed the co-authorship analyses by VOSviewer; different collaboration groups are represented by different colors. The co-authorship network is shown in Figure 6 and Figure 7. The VOSviewer presented the first author of the 6006 veterinary education publications showing the co-authorship networks in Figure 6, and the 677 veterinary communication education publications showing its co-authorship networks in Figure 7.

## 4. Discussion

Our results provide a fresh perception of the global view of the progressive paths for veterinary education publications (VEPs) and veterinary communication education publications (VCEPs). Over the past twenty years, the number of both VEPs (*n* = 6006, mean = 273.0 publication/year) and VCEPs (*n* = 677, mean = 30.8 publication/year) research publications per year have continued to increase. We found that the increase in publications reveals several interesting points. Firstly, there are two turning points found in the publication trend in the VEPs. Before 2016, there were few related publications, i.e., less than 300 publications per year. Additionally, since 2020, there has been a substantial increase, especially in 2021, in the number of publications that was more than 600. Secondly, veterinary communication education publications (VCEPs) accounted for about 12% to 14% of veterinary education publications (VEPs) from 2000 to 2019, and reached the highest point (15%) in 2021. Thirdly, the growth rate of research publications in veterinary communication education (from 9 in 2000 to 107 publications in 2021, with an increasing rate of 1189%) in recent decades was greater than that of veterinary education (from 101 to 684 publications with 577%). Especially in 2005–2008, 2011–2013, 2016–2017, and 2018–2021, the growth rate of journal publications in veterinary communication education showed an upward trend. From all these results, we can realize more and more researchers in the field of veterinary education focus on the issue of communication education.

This outcome reveals a 677.2% growth in publications in the last 22 years and an even higher growth (901.8%) in institutions. In relation to the author-affiliated institutions that published the 6006 veterinary education publications (VEPs), we found that more and more institutions have been actively involved in this field in the last two decades. There were 101 publications by 111 institutions (1.10 institutions per publication) in 2000, and 684 publications by 1001 institutions (1.46 per publication) in 2021, with a 32.7% increase rate.

We found an interesting point that all the top 10 highly cited publications were published between 2001 to 2016 in veterinary education publications (*n* = 6006) and between 2002 to 2017 in veterinary communication education publications (*n* = 677), separately. We found another interesting result that determined that this publication outcome fits the Pareto principle [35], which states that 80% of consequences come from 20% of causes (the “vital few”). For the total number of publications for veterinary education, 130 journals (14.9%) published 80.4% of the publications. Additionally, the results even revealed an extreme case in the Pareto principle: the top 10 journals (1.1% of 872 journals) published 40.63% of the total publications. This phenomenon is also shown in the subset of veterinary communication education publications.

There is also an interesting point obtained from our results. When comparing the top ten top citations with the top ten journals, we found that the JAVMA (Journal of the American Veterinary Medical Association, listed as the second highest publishing Journal shown in Table 3) has three highly cited publications by Shaw et al. (2004), Coe et al. (2007), and Coe et al. (2008) [14,15,16]. All these three highly cited works are related to the communication and relationship between the owners of the company animals and the veterinarians, with a focus on medical expenses and trust relationships. There are two highly cited publications by Jansen et al. (2010) [8] and Ritter et al. (2017) [9], which are from the Journal of Dairy Science (which is ranked eighth in publication quantities as shown in Table 3) and one highly cited publication by Garforth et al. (2013) [10] from Preventive Veterinary Medicine related to the communication and relationship between the owners of the economic animals and the veterinarians with a focus on animal health status, the prevention of infectious diseases, and prevention and control, respectively. However, we also noticed that there are highly cited works published by the journals that are not in the top 10 publishing rank list. For example, there were 2 highly cited publications by Gardner et al. (2006) [17] and Kristensen and Jakobsen (2011) [12] from the New Zealand Veterinary Journal, listed as 22nd (publication number 38, 0.63%), that focusing on the better communication skills of veterinarians would decrease the workplace stress of veterinarians and help to solve the dairy farmers’ management-decision challenges, respectively. All these reveal the importance of the dilemmas encountered in both the workplaces and field with insights into the unmet needs.

There were some limitations to this study. We searched the WoSCC database due to its high-quality database and peer-reviewed quality research publications, which does not include popular sciences or newspapers. Furthermore, we only included research publications from 2000 to 2021, and the limited number of search terms was another potential limitation. There were also limitations concerning the analytic tool. Although the VOSviewer is a free software and well applied in bibliometric analysis, which can recognize the networks among countries, organizations, and authors, the country names were not shown as capitalized, and the selection bias of VOSviewer underestimated the academic influence of authors due to VOSviewer only recognizing the first author.

This study delivered key characteristic information of publications in VEPs and VCEPs over the last 21 years. In addition to the traditional bibliometric descriptive analysis, we are inspired by reviewers and suggest that future studies could consider expanding the horizons on traditional bibliometric research perspectives to include inferential statistics to explore the differences significances among groups.

## 5. Conclusions

In conclusion, we found that the publication quantity has been increasing at a sharp slope rate over the last twenty years, which indicates that both veterinary education research and veterinary communication research have been in growing stages, and will attract increased attention in the future. In the veterinary education field, the United States published the most literature, followed by the United Kingdom, Australia, Canada, and Germany. The Journal of Veterinary Medical Education accounts for the largest proportion of veterinary education publications, followed by the Journal of the American Veterinary Medical Association (JAVMA), Veterinary Record, Animals, and Preventive Veterinary Medicine. Our bibliometric study provides unique information with respect to the high volume and variety of published data within the field of veterinary communication education, and it will be helpful for researchers who focus on this research field to acquire useful information about veterinary communication education. It also identifies collaborations among fields of research, researchers, and institutions useful in facilitating potential venues for future research and knowledge frontiers in the veterinary education research field.

## Figures and Tables

**Table 1:** The top 10 highly cited publications in veterinary education (VEPs) from 2000 to 2021.

Rank	Title	Times Cited *	Authors	Publication Title	Year	Type
1	2016 Guidelines of the American Society of Mammalogists for the use of wild mammals in research and education	1088	Sikes, R.S.	Journal of Mammalogy	2016	Article
2	Food-borne diseases—The challenges of 20 years ago still persist while new ones continue to emerge	615	Newell, D.G.; Koopmans, M.; Verhoef, L.; Duizer, E.; Aidara-Kane, A.; Sprong, H.; Opsteegh, M.; Langelaar, M.; Threfall, J.; Scheutz, F.; van der Giessen, J.; Kruse, H.	International Journal of Food Microbiology	2010	Article; Proceed-ings Paper
3	Global burden of Human Brucellosis: A Systematic Review of Disease Frequency	362	Dean, A.S.; Crump, L.; Greter, H.; Schelling, E.; Zinsstag, J.	Plos Neglected Tropical Diseases	2012	Article
4	Refugia—overlooked as perhaps the most potent factor concerning the development of anthelmintic resistance	362	Van Wyk, J.A.	Onderstepoort Journal of Veterinary Research	2001	Review
5	Fifteen years after Wingspread—Environmental endocrine disruptors and human and wildlife health: Where we are today and where we need to go	349	Hotchkiss, A.K.; Rider, C.V.; Blystone, C.R.; Wilson, V.S.; Hartig, P.C.; Ankley, G.T.; Foster, P.M.; Gray, C.L.; Gray, L.E.	Toxicological Sciences	2008	Review
6	The FAMACHA((c)) system for managing haemonchosis in sheep and goats by clinically identifying individual animals for treatment	280	Van Wyk, J.A.; Bath, G.F.	Veterinary Research	2002	Review
7	Multiple membership multiple classification (MMMC) models	231	Browne, W.J.; Goldstein, H.; Rasbash, J.	Statistical Modeling	2001	Article
8	Let’s get physical: Advantages of a physical model over 3D computer models and textbooks in learning imaging anatomy	207	Preece, D.; Williams, S.B.; Lam, R.; Weller, R.	Anatomical Science Education	2013	Article
9	Role of pet dogs and cats in the transmission of helminthic zoonoses in Europe, with a focus on echinococcosis and toxocariasis	202	Deplazes, P.; van Knapen, F.; Schweiger, A.; Overgaauw, P.A.M.	Veterinary Parasitology	2011	Article
10	Aquaculture as yet another environmental gateway to the development and globalization of antimicrobial resistance	192	Cabello, F.C.; Godfrey, H.P.; Buschmann, A.H.; Dolz, H.J.	Lancet Infectious Diseases	2016	Article

* Number of times cited is counted from the citation in WoSCC.

**Table 2:** The top 10 highly cited publications in veterinary communication education (VCEPs) from 2000 to 2021.

Rank	Title	Times Cited *	Authors	Publication Title	Year	Type
1	Debunking the myth of the hard-to-reach farmer: Effective communication on udder health	129	Jansen, J.; Steuten, C.D.M.; Renes, R.J.; Aarts, N.; Lam, T.J.G.M.	Journal of Dairy Science	2010	Article
2	Work-related stress in the veterinary profession in New Zealand	119	Gardner, D.H.; Hini, D.	New Zealand Veterinary Journal	2006	Article
3	Determinants of farmers’ adoption of management-based strategies for infectious disease prevention and control	118	Ritter, C.; Jansen, J.; Roche, S.; Kelton, D.F.; Adams, C.L.; Orsel, K.; Erskine, R.J.; Benedictus, G.; Lam, T.J.G.M.; Barkema, H.W.	Journal of Dairy Science	2017	Review
4	Description of the behavior of domestic dog (Canis familiaris) by experienced and inexperienced people	112	Tami, G.; Gallagher, A.	Applied Animal Behavior Science	2009	Article
5	Farmers’ attitudes to disease risk management in England: A comparative analysis of sheep and pig farmers	110	Garforth, C.J.; Bailey, A.P.; Tranter, R.B.	Preventive Veterinary Medicine	2013	Article
6	A focus group study of veterinarians’ and pet owners’ perceptions of the monetary aspects of veterinary care	97	Coe, J.B.; Adams, C.L.; Bonnett, B.N.	Journal of The American Veterinary Medical Association	2007	Article Proceed-ings Paper
7	A focus group study of veterinarians’ and pet owners’ perceptions of veterinarian–client communication in companion animal practice	95	Coe, J.B.; Adams, C.L.; Bonnett, B.N.	Journal of The American Veterinary Medical Association	2008	Article
8	What can veterinarians learn from studies of physician–patient communication about veterinarian–client–patient communication?	84	Shaw, J.R.; Adams, C.L.; Bonnett, B.N.	Journal of The American Veterinary Medical Association	2004	Article
9	Challenging the myth of the irrational dairy farmer; understanding decision making related to herd health	83	Kristensen, E.; Jakobsen, E.B.	New Zealand Veterinary Journal	2011	Review
10	Control of foot and mouth disease: lessons from the experience of the outbreak in Great Britain in 2001	79	Scudamore, J.M.; Harris, D.M.	Revue Scientifique Et Technique De L Office International Des Epizooties	2002	Article

* Numbers of times cited are counted from the citation in WoSCC.

**Table 3:** The top 10 productive journals for veterinary education publications (VEPs) from 2000 to 2021.

Rank	Journals	Publications Number * (%)	Impact Factor **	Category	Ranking
1	Journal of Veterinary Medical Education	1191 (19.83%)	1.027	Education, Scientific Disciplines Veterinary Sciences	36/44 96/146
2	Javma Journal of The American Veterinary Medical Association	263 (4.38%)	1.936	Veterinary Sciences	51/146
3	Veterinary Record	257 (4.28%)	2.695	Veterinary Sciences	25/146
4	Animals	145 (2.41%)	2.752	Agriculture, Dairy and Animal Science	13/63
5	Preventive Veterinary Medicine	129 (2.15%)	2.67	Veterinary Sciences	27/146
6	Revue Scientifique Et Technique Office International Des Epizooties	118 (1.97%)	1.181	Veterinary Sciences	90/146
7	Frontiers in Veterinary Science	114 (1.90%)	3.412	Veterinary Sciences	9/146
8	Veterinary Surgery	77 (1.28%)	1.495	Veterinary Sciences	74/146
9	Journal of Dairy Science	75 (1.25%)	4.034	Agriculture, Dairy and Animal Science Food Science and Technology	6/63 40/143
10	Australian Veterinary Journal	71 (1.18%)	1.281	Veterinary Sciences	86/146

* Total veterinary education publications (VEPs) were 6006, which were published in 872 journals from 2000 to 2021. ** Impact factors source are from the Journal Citation Reports.

**Table 4:** The top 10 productive journals for veterinary communication education publications (VCEPs) from 2000 to 2021.

Rank	Journals	Publications Number * (%)	Impact Factor **	Category	Ranking
1	Journal of Veterinary Medical Education	215 (31.76%)	1.027	Education, Scientific Disciplines Veterinary Sciences	36/44 96/146
2	Veterinary Record	32 (4.73%)	2.695	Veterinary Sciences	25/146
3	Frontiers in Veterinary Science	30 (4.43%)	3.412	Veterinary Sciences	9/146
4	Journal of The American Veterinary Medical Association	29 (4.28%)	1.936	Veterinary Sciences	51/146
5	Animals	21 (3.10%)	2.752	Agriculture, Dairy and Animal Science	13/63
6	Journal of Dairy Science	20 (2.95%)	4.034	Agriculture, Dairy and Animal Science Food Science and Technology	6/63 40/143
7	Preventive Veterinary Medicine	18 (2.66%)	2.67	Veterinary Sciences	27/146
8	Revue Scientifique Et Technique Office International Des Epizooties	12 (1.77%)	1.181	Veterinary Sciences	90/146
9	Veterinary Clinics: Small Animal Practice	12 (1.77%)	2.093	Veterinary Sciences	45/146
10	Australian Veterinary Journal	9 (1.33%)	1.281	Veterinary Sciences	86/146

* Total veterinary communication education publications (VCEPs) were 677, which were published in 163 journals from 2000 to 2021. ** Impact factors source are from the Journal Citation Reports.

**Table 5:** The top 10 productive countries for veterinary education publications (VEPs) and veterinary communication education publications (VCEPs) from 2000 to 2021.

Veterinary Education Publications (N = 6006)			Veterinary Communication Education Publications (N = 677)		
Rank	Country/Region	Publications Number (%)	Rank	Country/Region	Publications Number (%)
1	USA	2195 (36.55%)	1	USA	296 (43.72%)
2	ENGLAND	777 (12.94%)	2	ENGLAND	115 (16.99%)
3	AUSTRALIA	460 (7.66%)	3	CANADA	84 (12.41%)
4	CANADA	435 (7.24%)	4	AUSTRALIA	71 (10.49%)
5	GERMANY	294 (4.90%)	5	GERMANY	31 (4.58%)
6	SCOTLAND	225 (3.75%)	6	NETHERLANDS	31 (4.58%)
7	ITALY	209 (3.48%)	7	SCOTLAND	26 (3.84%)
8	BRAZIL	199 (3.31%)	8	ITALY	23 (3.4%)
9	NETHERLANDS	198 (3.30%)	9	NEW ZEALAND	18 (2.66%)
10	FRANCE	170 (2.83%)	10	BELGIUM	14 (2.07%)

## Data Availability

Data sharing not applicable.
